# A Review on Replacing Food Packaging Plastics with Nature-Inspired Bio-Based Materials

**DOI:** 10.3390/foods14101661

**Published:** 2025-05-08

**Authors:** Shengsi Hu, Lu Han, Chenfeng Yu, Leiqing Pan, Kang Tu

**Affiliations:** College of Food Science and Technology, Nanjing Agricultural University, Nanjing 210095, China; 2024108049@stu.njau.edu.cn (S.H.); 2022208020@stu.njau.edu.cn (L.H.); 2023108046@stu.njau.edu.cn (C.Y.); pan_leiqing@njau.edu.cn (L.P.)

**Keywords:** biomimetic design, biodegradable solutions, food packaging

## Abstract

Food packaging is critical to delaying food spoilage, maintaining food quality, reducing food waste, and ensuring food safety. However, the environmental problems associated with petroleum-based packaging materials have led to a search for sustainable alternatives. Bio-based materials are emerging as such alternatives, but they have limitations such as low mechanical strength and poor moisture resistance. Fortunately, nature’s insights guide solutions to these challenges, propelling the evolution of high-performance bio-based packaging. These new food packaging materials are characterized by impact resistance, superhydrophobicity, self-healing capabilities, dynamic controlled release, high mechanical strength, and real-time freshness monitoring. Nature-inspired strategies not only focus on enhancing material performance but also introduce innovative design concepts that effectively avoid the homogenization of food packaging and inspire researchers to develop diverse, cutting-edge solutions. Overcoming the existing problems of bio-based materials and endowing them with breakthrough properties are key drivers for their replacement of food packaging plastics. This review provides insights into the application of biomimetics in enhancing the functionality of bio-based materials and clearly articulates the key drivers for the replacement of plastic food packaging by bio-based materials. By systematically integrating existing research findings, this paper identifies the challenges facing nature-inspired food packaging innovations and points the way to their future development.

## 1. Introduction

Every year, approximately one-third of food is lost or wasted worldwide, amounting to an estimated value of $936 billion, an amount that excludes the accompanying social and environmental expenses [1,2]. Meanwhile, according to statistics from 2020, it is revealed that 3 billion individuals worldwide cannot afford nutritious meals, while 811 million people are struggling with hunger [3]. As the population continues to grow, the demand for food continues to climb, and these losses would be sufficient to alleviate malnutrition for about one-eighth of the world’s population and effectively address future increases in food demand. Reducing food loss and waste is therefore pivotal to maintaining global food security. Food packaging plays a key role in isolating moisture and reducing microbial contamination, which has a significant impact on reducing food spoilage and waste [4,5]. In the current food packaging sector, petroleum-based plastics are widely used due to their low production cost, lightweight, flexible, and durable properties. However, these plastic packaging materials are difficult to degrade and pose a serious threat to the natural environment. In addition, more and more studies reveal that microplastics, which are potentially hazardous to human health, including the possibility of gastrointestinal diseases, respiratory problems, cancers, and infertility [6,7,8,9,10].

**Figure 1:**
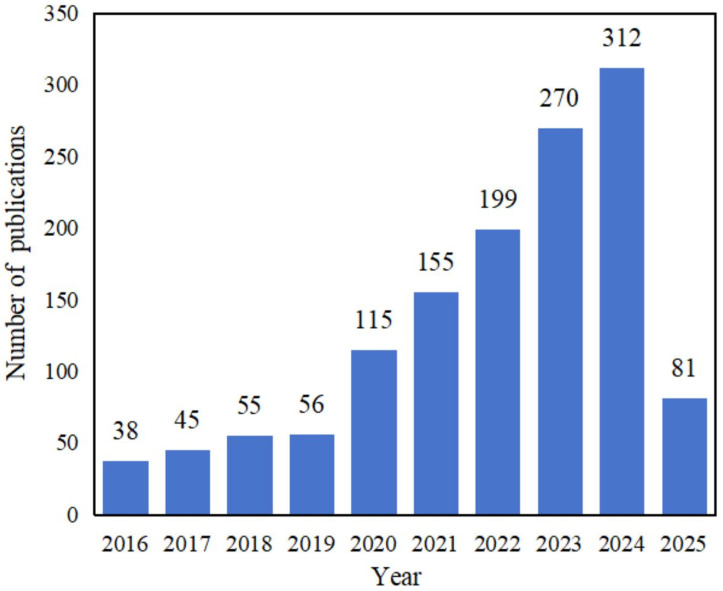
Trends of research article publications in the field of alternative plastic packaging for food in the last decade (data from the Web of Science with themes of “alternative plastic packaging for food”).

In response to the environmental and health risks associated with petroleum-based plastic packaging, researchers are increasingly focusing on the development of sustainable alternatives for food packaging, as evidenced by the growing number of papers in this field (Figure 1). Among the various solutions, bio-based materials are particularly promising candidates due to their renewable origin, biodegradability, and safety. Well-known examples include cellulose derivatives (e.g., cellulose acetate, methyl cellulose, hydroxypropyl cellulose, carboxymethyl cellulose), polylactic acid, chitosan, and starch. However, bio-based materials encounter substantial challenges in packaging applications owing to their inherent limitations, including single-functionality, poor solubility, weak mechanical properties, moisture sensitivity, brittle behavior, and highly crystalline structures, which collectively impede their practical applications [11]. For instance, polylactic acid suffers from high brittleness, low toughness, hydrophobicity, and slow biodegradation, compounded by cost and processing constraints. High-amylose starches, while mechanically improved, exhibit extrusion difficulties from elevated gelatinization temperatures and viscosity, while thermoplastic starches struggle with moisture susceptibility and inferior mechanical/thermal performance. Additionally, protein-based membranes show poor water vapor barriers due to their hydrophilic characteristics, making them vulnerable to environmental moisture [12,13,14,15]. In order to solve these problems of bio-based materials, researchers usually use blending [16,17], adding metal nano-oxides [18,19], and modification [20]. However, the blending method has a relatively limited range of material options, which tends to result in highly similar products. This homogeneity precisely stifles innovation. Additionally, the improvement in mechanical properties through blending is limited; the introduction of metal nano-oxides may bring potential safety hazards [21,22], and the modification method is relatively complex and consumes a large amount of chemical reagents, which may have a negative impact on the environment [23]. Therefore, there is an urgent need for new technologies and methods to promote the application of bio-based materials in the food field.

The concept of “learning from nature” has made significant progress in many fields (e.g., desalination [24], soft robotics [25], flight design [26], materials science [27], etc.). However, learning from nature has been relatively underutilized in the field of food. More importantly, based on the current scientific advancements, it is hoped that deficiencies in food bio-based materials, such as their weak mechanical properties and poor barrier capabilities, can be solved through inspiration drawn from nature. Drawing inspiration from nature not only opens up new design paradigms but also directly empowers material development with breakthrough bio-inspired properties like self-healing. Solving the challenges inherent in bio-based food packaging, enhancing its performance, and endowing it with breakthrough functionalities such as self-healing capabilities are critical drivers for the substitution of conventional plastic packaging in the food industry. Although established studies have initially explored the potential of biobased materials, the strategic value of natural inspiration in the development, design, and application of biobased materials has not been fully appreciated. Therefore, this review aims to fill the current research gap and deeply explore the strategic value of nature inspiration in bio-based materials development. Specifically, this paper analyzes in detail how biomimetic strategies can enhance the comprehensive performance of biobased materials, focusing on the development of biobased packaging materials with excellent properties such as impact resistance, superhydrophobicity, gas regulation ability, self-repairing, real-time freshness intelligent monitoring, and high mechanical strength, as well as pointing out the driving force for their replacement of plastic food packaging. It also critically examines the limitations of current research and outlines the future direction of nature-inspired innovation in food packaging.

## 2. Anti-Mechanical Damage Packaging

**Figure 2:**
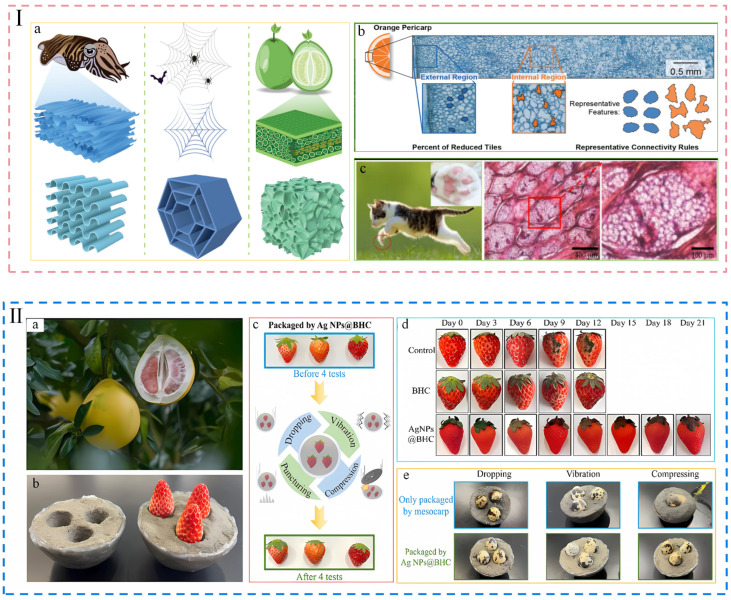
(**I**) (**a**) The process of extracting and synthesizing the design of cushioning structures of cuttlefish bones, spider webs, and grapefruit peels [39]; (**b**) cross-sectional images of orange peels, which provide practical structural references for tile classification and the definition of connectivity rules [40]; (**c**) the three-layer structure of a cat’s paw (epidermis, dermis, and subcutaneous layer in that order), with the subcutaneous layer being the main energy-absorbing layer [41]. (**II**) (**a**) Photographs of grapefruit layering; (**b**) the composite material wrapped strawberries; (**c**) Performance of composite sponges in resistance to injury tests; (**d**) changes in the appearance of strawberries wrapped in composites during a 21-day storage test; (**e**) changes in quail eggs packed in composite sponge after damage resistance test [42]. The above pictures have been reproduced with permission.

Fruits and vegetables are vulnerable to mechanical damage from various physical factors during harvesting, transportation, processing, and storage. These damages typically arise from scuffs and scratches incurred during manual harvesting, collisions with harvesting machinery, vibrations during transit, and the compressive forces from stacking. The repercussions of mechanical damage on fruits and vegetables are significant: physiologically, it results in a marked elevation in respiratory rates, accelerated enzymatic browning, and a substantial release of ethylene [28,29]. These detrimental impacts are reflected to varying extents in bananas [30], peaches [31], apples [32], as well as other types of fruits and vegetables. Economically, it diminishes the quality of produce and amplifies supply chain losses; and from a safety standpoint, it may facilitate fungal colonization and toxin production, leading to issues such as cellular degradation and ecological contamination. However, traditional plastic packaging struggles to provide effective protection. To mitigate the issue of mechanical damage to fruits and vegetables during transportation and storage, researchers are actively developing packaging materials that are highly effective in resisting such damage. For example, the porous foam crafted from corn alcohol-soluble protein and soybean oil polyol exhibited excellent protective properties in the bruise test. While control tomatoes exhibited cracks after just 3 collisions, tomatoes packaged in this foam required 73 collisions before any cracks appeared, demonstrating its remarkable ability to safeguard the tomatoes against mechanical impact [33]. However, the design of packaging materials with high cushioning performance is not limited to foam structures. Examples include the multi-layered keratinous claws of cats, the turgor pressure-regulated peel of oranges, the “wall-diaphragm” architecture in squid beaks, and the gradient porosity of grapefruit mesocarp, all of which exhibit remarkable efficiency in force distribution [34,35,36,37,38,39,40,41]. To be specific, using squid bone (S-shaped structure), spider web (polygonal web structure) and grapefruit skin (porous structure) (Figure 2Ⅰ(a))as biological prototypes, the researchers used silica gel (to prepare the S-shaped spider web structure, and polyurethane foam to fill in the voids to mimic the porous structure) to prepare a bio-inspired structural composite material, which can reduce the peak impact force by 3.5 times under impact loading and performs well in heel and egg [39]. Another researcher, by analyzing the cross-section image of an orange peel (Figure 2Ⅰ(b)), discretized the irregular biological structure into a series of building blocks and the rules for connecting these blocks. By analyzing the types of building blocks and the rules for connecting them through statistical methods, a synthetic material with the same distribution was generated. The results show that the material has similar spatially varying stiffness as the orange peel, good energy absorption ability, and local deformation control, which successfully solves the problem of replicating the irregular biostructures and realizing similar mechanical properties [40]. Similarly, the cat’s claw has a three-layered structure of epidermis, dermis and subcutis (Figure 2Ⅰ(c)), and the softer subcutis is the main energy-absorbing layer, which contains elastic collagen fiber bundles to form a multiscale crisscross network scaffolding, and the porous framework defines two topological chambers containing adipose tissue, with the elastic fiber network providing the mechanical architecture and integrity, and the adipose tissue being the main cause of energy dissipation [41]. Composites inspired by this design also have good cushioning capacity. Figure 2II demonstrates the specific application of representative bio-inspired designs in strawberry packaging, showcasing their practical utility. Thus, Figure 2I provides a biological reference for researchers seeking to develop resistance to mechanical damage, while Figure 2II illustrates the targeted application of these concepts to food products, clearly demonstrating the advancement from natural inspiration to practical application.

As shown in Figure 2II, the researchers drew inspiration from the layered structure of grapefruit peels (a) to develop composites (Ag NPs @ BHC) that primarily mimic the mesocarp and exocarp to enhance cushioning and freshness preservation of the fruit. The mesocarp is designed as a multilayered honeycomb sponge, which is soft and elastic enough to protect the fruit by absorbing external impacts, while the exocarp consists of a dense, filamentous hydrogel, which is puncture-resistant and disperses mechanical stresses. The layers are firmly bonded by hydrogen bonds to maintain structural integrity. Tests showed that this composite material had a significant protective effect on strawberries, enabling them to pass four consecutive tests, including shock, vibration, compression, and perforation (b,c) with intact morphology and no visible damage, and extending the shelf life of the strawberries to 21 days (d), compared to 9 days for the control group [42]. It is worth emphasizing that in another set of tests, the quail eggs in the control group were severely broken after the drop and compression tests, but remained intact after the vibration test. However, the quail eggs filled with Ag NPs @BHC remained undamaged after all tests, demonstrating their excellent resistance to mechanical damage (e). Also of note, researchers created a soft, elastic holocellulose sponge with a layered structure using microcrystalline cellulose as a nanofiller. The sponge, containing macropores and small cotton-filled pores, exhibited elasticity due to sequential strain absorption by microcrystalline cellulose. It showed low stress (48.40 kPa at 70% strain) and minimal permanent deformation (18.04% after 4000 presses). This sponge effectively protected strawberries from damage during dropping or shaking tests [43]. Other researchers have also mimicked the spongy structure to achieve good results in food resistance to mechanical damage [44,45].

## 3. Application of Superhydrophobicity in Food Packaging

In the field of food packaging, the application of superhydrophobic technology is receiving increasing attention, and the superhydrophobic properties of swan feathers and lotus leaves in nature provide a valuable source of inspiration for design innovation in this field. Swan feathers are highly water-resistant due to their complex multilayered structure, tiny hooked micron-sized protrusions, and nano-sized scales, as well as a layer of prenatal oils on the surface, which together form an impenetrable waterproof barrier [46]. Similarly, the lotus leaf cleverly utilizes its densely packed microscopic papillae and the wax layer covering it to create a nanoscale air film that makes it difficult for water droplets to stay on the surface, thus achieving superior superhydrophobicity [47]. The introduction of bionic superhydrophobic structure endows food packaging materials with multifaceted functional innovations: through the mechanism of surface wettability regulation, it can not only inhibit microbial proliferation, maintain the dry state of food products and significantly extend the shelf-life, but also reduce the wastage due to the adhesion of food liquids, and at the same time provide technical support for the development of environmentally friendly paper-based packaging materials. This section will systematically analyze the innovative applications of the technology in three aspects: extension of shelf life due to moisture resistance, realization of biomimetic structures in biodegradable straws, and the correlation between moisture resistance and reduction in food waste.

### 3.1. Superhydrophobic-Preservation

**Figure 3:**
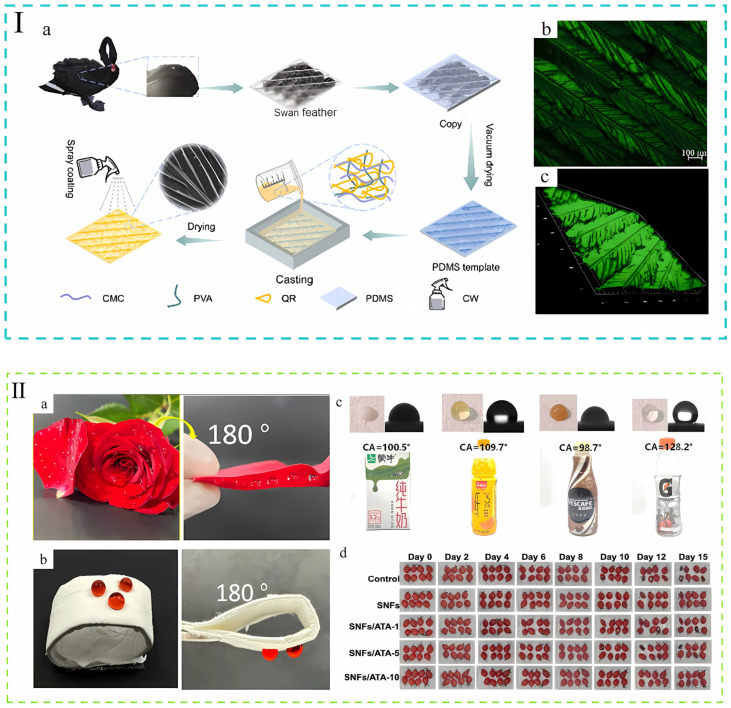
(**I**) (**a**) Swan feather-inspired composite film preparation process. (**b**,**c**) Laser confocal microscopy images of composite membranes with swan feather surface texture [48]. (**II**) (**a**) Water adhesion and hydrophobicity of rose petals. (**b**) Schematic representation of the water adhesion and hydrophobicity of the bionic composite membrane (SNF/ATA-10). (**c**) Water contact angles of bionic films with common commercial beverages, from left to right, plain milk, orange juice, coffee, and electrolyte water. (**d**) Effect of different packaging on the freshness of cherry tomatoes [49]. The above pictures have been reproduced with permission.

Moisture is a key factor in promoting rapid microbial reproduction, which can accelerate the spoilage process of food and thus pose a potential threat to consumer health. At the same time, moisture also makes crunchy foods (e.g., potato chips) soft, severely affecting their texture, shortening their shelf life, and increasing food waste. Nature offers a distinctive approach guidance to resolving this predicament. In order to more visually represent the hydrophobic properties and understand the hydrophobic properties, Figure 3Ⅰ(a,b) provides the hydrophobic film preparation process inspired by swan feathers and the surface texture of the film [48] and Figure 3II represents the rose petal-inspired bionic film (a,b), as well as the film’s hydrophobicity to various liquids (c) and superior preservation performance, evidenced by extending cherry tomato shelf life to 15 days (d) [49]. In addition, Table 1 details specific examples of nature’s contribution to the development of hydrophobic food packaging. It is worth noting that food packaging films without specialized hydrophobic treatment typically exhibit a water contact angle (WCA) ranging from 80° to 95° [50,51,52,53,54] (a WCA below 90° indicates hydrophilicity). Interestingly, among the many methods for preparing hydrophobic surfaces, polydimethylsiloxane stands out due to its frequent mention in several papers, which is a testament to the broad applicability of this method.

Despite the progress in research on biomimetic superhydrophobic materials, their industrialization still faces two major challenges: high cost and preparation complexity, including the reliance on precision lithography equipment and expensive reagents, which restricts large-scale production; and insufficient durability, as the surface structure is susceptible to damage by physical injury and environmental factors (e.g., temperature, pH, and ultraviolet rays), and the existing stability assessments are mostly based on short-term experiments, which are difficult to meet the actual industrial needs [55]. In addition, most of the existing studies focus on hydrophobicity enhancement, but lack practical relevance to the extension of shelf life or maintenance of food quality, and need to further elucidate the direct correlation between the increase in hydrophobicity angle and freshness preservation effect.

**Table 1:** Nature-inspired superhydrophobic food packaging.

Bionic Object	Main Material	Main Conclusion	References
Swan feather	Carboxymethyl cellulose, polyvinyl alcohol, Brazilian carnauba wax	The film has a water contact angle of 138°, similar to feathers, and extends pork shelf life to 5 days, better than the 2 days of regular packaging.	[48]
Rose petal	Starch nanofiber membrane, acylated tannins	The film had a water contact angle of approximately 134.1° and was also able to extend the shelf life of cherry tomatoes to 15 days compared to 8 days for the control.	[49]
Duck feather	Carboxymethyl cellulose, gelatin, candelilla wax	The film features a water contact angle of 142.57° and increases the shelf life of beef to 5 days, providing an additional 2 days compared to standard polyethylene packaging.	[56]
Lotus leaf	Konjac glucomannan, polylactic acid, tea polyphenols	The fiber boasts a water contact angle exceeding 150°, enabling self-cleaning properties and prolonging the shelf life of cabbage to 6 days and potatoes to 10 days.	[57]
Lotus leaf	Nanoparticles of chitosan, sodium alginate, and zeinolysin	The water contact angle of the outer layer of the membrane exceeds 130° and significantly reduces the rate of deterioration of apple rosettes.	[58]
Lotus leaf	Soybean polysaccharide, carnauba wax	The film has a water contact angle of 157.2°, and freshness tests have shown that grapes packed in this film can still be eaten at 7 days.	[59]
Lotus leaf	Nano-silica, chitosan, acrylic acid, rosin	Paper with this coating has a water contact angle of 155.8° and an oil contact angle of 92°. In a 6 h storage test, the residue rate of water/soda/tea/milk/coffee was only 0.5–2.0%, significantly lower than that of commercial paper cups, which effectively reduces liquid residue.	[60]
Mussel	Chitosan, curcumin, hydrophobic SiO_2_	The composite film, exhibiting a water contact angle of 130.43°, extends the shelf life of pork by two days when stored at both 25 °C and 4 °C.	[61]
Taro leaf	Alkyl ketene dimer, cellulose nanofiber, cellulose powder, TiO_2_ nanoparticles	The superhydrophobic coating creates a waterproof barrier by boosting the surface hydrophobicity angle (e.g., filter paper up to 173°), keeping tomatoes fresh for 14 days, while uncoated ones mold.	[62]

### 3.2. Superhydrophobic—Paper Straws

**Figure 4:**
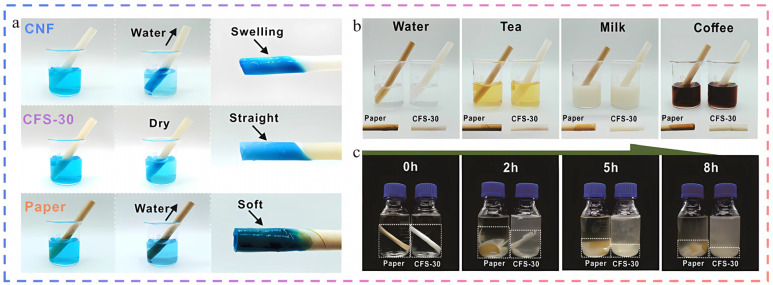
(**a**) Schematic of CNF straws, CFS straws and commercial paper straws after simultaneous immersion in aqueous methyl blue; (**b**) schematic of CFS straws and commercial paper straws after immersion in commercial beverages such as water, tea, milk, and coffee at 0 °C and 65 °C for 30 min, respectively; (**c**) schematic of the dissolved state of CFS straws and commercial paper straws after soaking in NaOH/urea solution, freezing and thawing four times [65]. The above pictures have been reproduced with permission.

Plastic straws, as an accessory to plastic food packaging, also pose significant environmental hazards. They share the same issues of being difficult to degrade and prone to releasing microplastics, which can have devastating effects on the environment and human health. In response to these concerns, traditional paper straws have emerged as a promising alternative due to their biodegradability. However, paper straws also face challenges, particularly their poor water-blocking ability, which can lead to quick soaking and disintegration [63,64]. To solve this problem, inspired by the hydrophobicity of sugarcane peels, researchers modified cellulose nanofibers with stearic acid to develop a green composite straw (CFS) that combines superhydrophobic properties (water contact angle as high as 153°), high mechanical strength (tensile strength up to 67.15 MPa), and good environmental recyclability, which, compared with commercial paper straws, in terms of water resistance, mechanical properties, and dissolution rate [65]. Figure 4 demonstrates the excellent stability of the CFS straws: after immersion in methylene blue solution, the CFS straws remained intact, while the other samples swelled and softened (a). In addition, when the CFS straws were immersed in water of different temperatures or in different beverage environments, their shape remained unchanged without swelling or bending, confirming their usefulness (b). After treatment, the CFS straws produced very little debris after 8 h of dissolution. In contrast, commercial paper straws dissolved more slowly, initially delaminating but leaving larger fragments; 52.87% of their mass was still in fragments after 8 h. These results indicate that CFS straws have a higher safety and recycling potential than commercial paper straws (c).

### 3.3. Superhydrophobicity—Reducing Food Waste

Plastic packaging for food is well-known for being slow to decompose, resulting in ongoing concerns about environmental pollution. It releases microplastics into the ecosystem, posing a long-term threat to marine life and natural habitats. Additionally, the surface chemistry of these plastics often results in significant food residue, particularly in viscous products like yogurt and cooking oil. This residue not only represents a waste of food resources but also complicates efforts to recycle the packaging materials, further exacerbating environmental issues and contributing to resource wastage.

**Figure 5:**
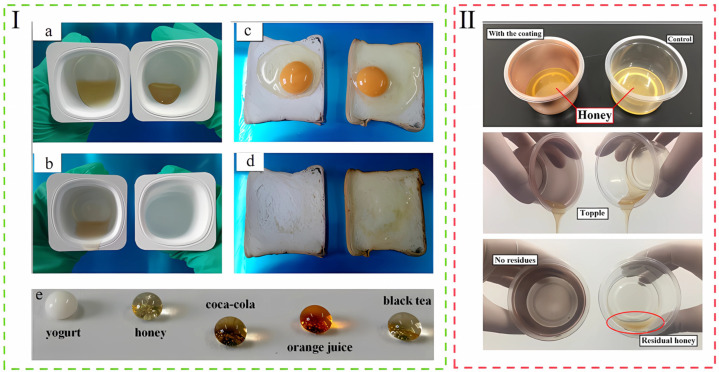
(**I**) (**a**) Uncoated cups containing honey (**left**) and cups with superhydrophobic coating (**right**) before pouring. (**b**) Photographs of uncoated cups containing honey (**left**) and cups with superhydrophobic coating (**right**) after pouring. (**c**) Photographs of sticky raw eggs on uncoated bread (**right**) and sticky raw eggs on bread with superhydrophobic coating (**left**) before pouring. (**d**) Photographs of sticky raw eggs on uncoated bread (**right**) and sticky raw eggs on bread with superhydrophobic coating (**left**) after pouring. (**e**) Effect of coatings on the repulsion of various droplets [67]. (**II**) Photographs of honey in a control cup (**right**) and a cup coated with a superhydrophobic coating (**left**): (**top**) before pouring; (**middle**) during pouring; (**bottom**) after pouring [68]. The above pictures have been reproduced with permission.

In order to solve these issues, Li and the others prepared bio-inspired edible superhydrophobic interfaces using edible beeswax, gum Arabic, and gelatin through a simple microemulsion spraying method. The interface mimics the surface of a lotus leaf and significantly reduces the residue of high value-added liquid food products in containers. With a contact angle greater than 150°, enhanced adhesion and durability, and non-toxicity and no volatile organic solvent residue, it provides new ideas for food packaging materials [66]. Similar coatings made of small candle tree wax and rice bran wax also showed extreme superhydrophobicity with contact angles higher than 150°, and were effective on a wide range of non-Newtonian viscous liquids. Remarkably, when applied to polypropylene containers, the coatings retained their excellent superhydrophobicity even after being subjected to immersion in hot aqueous solutions (70 °C) or after undergoing up to 1200 repeated bending tests, demonstrating exceptional heat resistance and outstanding flexibility. This finding further expands the application scope of superhydrophobic coatings in food packaging, especially in scenarios that need to withstand complex physical environmental changes [67]. The practical testing effects of this coating, which vividly demonstrate its capability to repel liquids like honey (a,b), egg wash (c,d), and others in challenging conditions (e), are shown in Figure 5Ⅰ. Additionally, Zhang et al. developed a heat-resistant, edible superhydrophobic coating made of beeswax and coffee lignin that mimics leaf structures. As demonstrated in Figure 5II, the uncoated containers had more than 15% and 18% residue in honey in the tests, while the coated containers had almost negligible residue, indicating that the coatings are very effective in reducing food waste [68].

In conclusion, biomimetic hydrophobic coatings exhibit dual benefits: they effectively reduce liquid food residue, thereby mitigating food waste, and, thanks to ongoing research advancements, they hold the promise of being integrated into the development of paper-based food containers, offering a viable alternative to plastic containers within the food industry.

## 4. Air-Conditioned Packaging

**Figure 6:**
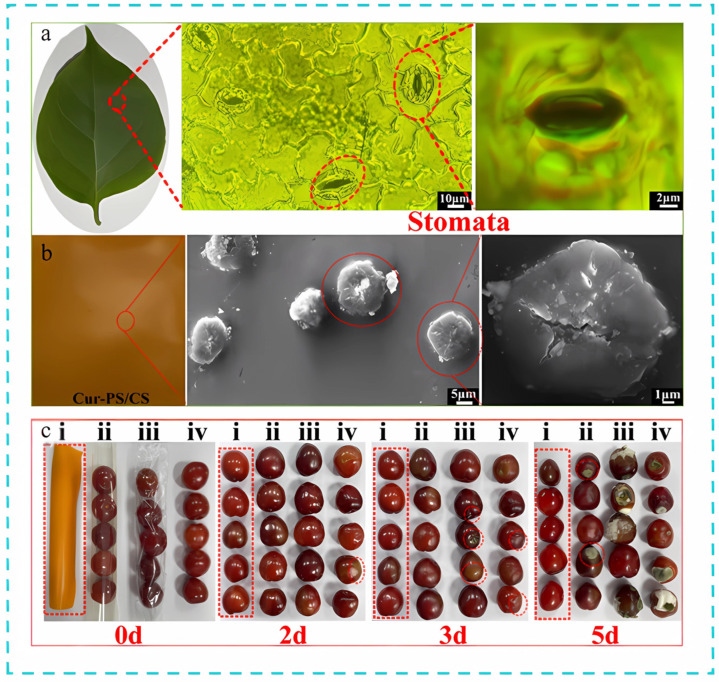
(**a**) Physical images of leaves and microscopic images of leaf stomata. (**b**) The surface SEM images of Cur-PS/CS. (**c**) Cherry preservation characteristics of different films at different times (2–5 d) [70]: (i) Cur-PS/CS, (ii) Chitosan (CS), (iii) commercial PE film, (iv) control group. The red box emphasizes the freshness preservation of the Cur-PS/CS group.

Oxygen, a primary cause of food oxidation, presents significant preservation challenges. Researchers have found inspiration in plant stomata, which are specialized structures that not only control water transpiration but also precisely regulate gas exchange. By selectively absorbing carbon dioxide and releasing oxygen, maintained through unique pore-size regulation and gas-selective permeability, plant stomata provide valuable insights for the development of packaging materials aimed at preserving fruits and vegetables. Inspired by this, researchers have developed a series of functional packaging materials with gas-selective permeation. For example, the highly selective permeation of carbon dioxide and oxygen was achieved by simulating the stomatal structure of plants with a hybrid membrane designed by mixing polylactic acid, chitosan porous microspheres, and tannic acid into a violet gel membrane. Specifically, the carbon dioxide/oxygen selectivity of the hybrid membrane reached 8.6, which was 1.8 times that of the pure shellac membrane, a value significantly better than that of traditional packaging materials, and showed excellent freshness preservation performance for five fruits with different respiratory metabolisms, among which oranges were preserved freshness for up to 45 days. The authors also investigated the reusability of the hybrid membrane packaging box. The results showed no significant change in the weight and edibility of cherries after three cycles of recycling [69]. Similarly, the researchers designed a curcumin porous starch/chitosan bionic smart aeropack that demonstrated precise regulation of carbon dioxide and oxygen selectivity. Figure 6 shows the stomata on the leaf surface (a) and the bionic stomata inspired by this on the surface of the composite film (Cur-PS/CS) (b). In the preservation application as shown in Figure 6c, the cherries wrapped in this composite film still had good freshness and eating quality on the fifth day [70]. In addition, films inspired by the porous structure of leaves [71], as well as a wormwood hydrogel/chitosan hybrid film based on the stomatal mechanism of the purple gummy worm, “Purple Gum Blanket,” also showed optimized CO_2_ and O_2_ selectivity [72]. In a similar vein, the researchers solved the problem of high porosity in paper-based packaging by taking inspiration from eggshells and creating a “paper window” system to improve traditional paper packaging. It uses plain paper coated with a nano-chitosan/polyethylene glycol film to improve CO_2_/O_2_ selectivity (1.65–3.71), which extends lychee freshness by 8 days at a temperature of 25 °C, which is superior to that of plain packaging and provides new ideas for developing sustainable paper-based packaging that can help further replace food packaging plastics [73].

## 5. Controlled Release Packaging

Through adaptive evolution, plants have internalized stimulus-responsive volatile release mechanisms as survival strategies (e.g., Cestrum nocturnum precisely releases odorous substances through moisture gate stomata during high humidity conditions at night to attract nocturnal pollinators and ensure pollination efficiency; plants release volatile organic compounds (VOCs) after herbivory in order to induce systemic (defense responses to reduce further damage). Inspired by this bio-environment-response-survival paradigm, smart controlled-release packaging technology encapsulates antimicrobial agents in reactive carriers embedded in matrix films, enabling dynamic sensing of food spoilage cues (e.g., pH fluctuations or humidity migration gradients) and on-demand release of the active agent, thus extending shelf-life while accurately inhibiting the development of spoilage. Table 2 summarizes the most recent papers according to the different response objects (pH, humidity, light, temperature, and enzymes) of the controlled release package.

Controlled release packaging technology greatly reduces the ineffective release of antimicrobial agents and plays an important role in extending the shelf life of food products. However, controlled-release food packaging also faces some challenges: (1) Most research has focused on anti-bacterial, with less research on anti-viruses [74]. (2) Research on controlled release is simply limited to the laboratory, ignoring the complex environment in real food [75]. (3) Toxic reagents may be introduced during the process of making controlled-release packaging, posing a potential hazard to food safety. (4) Anti-microbial agents (e.g., silver ions, essential oils) embedded in the packaging may leach into the food, resulting in off-flavors, texture changes, or residue limit violations [76,77]. Together, these gaps create a risky game between innovation and safety.

**Table 2:** Current research on controlled release packaging.

Main Material	Response Object	Main Conclusion	References
Thymol, ZIF-8,κ-carrageenan, zein	pH	By 36 h, the cumulative release of thymol was approximately 92% at pH 4.5 and 39.3% at pH 7.4. When practically applied to freshness, the shelf life of blueberries was extended by 9 days.	[78]
Starch, polyvinyl alcohol, essential oil of clove, Trichoderma mycelium	pH	Trichoderma mycelium film reaches release equilibrium 33 h later than ordinary films. The film keeps shrimp quality stable for 8 days.	[79]
Gelatin, beta-cyclodextrin, oregano essential oil	pH	Tested at 105 °C, the cumulative release of essential oils in 12 h was only 15%, much lower than the unencapsulated 60%. This film can extend the shelf life of grass carp filets by 2–3 days when refrigerated at 4 °C.	[80]
Corn Alcohol Soluble Protein/β-Cyclodextrin-Metal–Organic Framework/carvacrol	humidity	Thymol-loaded ZIF-8-based films exhibited 96.3 ± 1.5% cumulative release at 100% RH (36 h) vs. 12.0 ± 0.8% at 43% RH, enabling 7-day preservation of strawberries at 21 °C/50% RH (vs. 3-day spoilage in controls).	[81]
Nanofiber film, zein, polyethylene oxide, thyme essential oil, sodium bicarbonate, citric acid	humidity	The film maintained 64% thymol release at 60 h under high humidity (vs. 25% under low humidity), sustaining superior strawberry preservation quality over 6 days compared to control films.	[82]
CeCDs, folic acid, ZnCl_2_	light	The hydrogel releases a high concentration of Zn^2+^ for more than 15 days, resulting in a sustained antimicrobial effect that effectively extends shelf life and ensures food quality by controlling microbial contamination when applied to cabbages, apples, and cooked meats.	[83]
Polyvinylidene fluoride, rose Bengal, N-N-dimethylformamide	light	Rose Bengal generates reactive oxygen species for sterilization when exposed to light and stabilizes the encapsulation to avoid reactive oxygen species leakage in the absence of light; the film extends the shelf life of pork at 4 °C by 3 days.	[84]
Polylactic acid, lemon essential oil, polyvinyl alcohol, poly(N-isopropylacrylamide)	temperature	The release rate of lemon essential oil was low at 20 °C for 24 h, and the release rate increased to 47.16% at 40 °C. In strawberry preservation experiments at 25 °C/80% RH and 35 °C/80% RH, the decay rate was significantly lower than the control.	[85]
Tea polyphenol, chitosan, polyaspartic acid, polyvinyl alcohol	Enzyme and pH	The nanofiber film achieved 56.22% cumulative release of lemon essential oil at pH 5.0 over 120 h, with a 25.31% enhancement in the presence of protease (1 mg/mL), and exhibited complete suppression of strawberry mildew growth for 6 days, significantly exceeding the 4-day mildew onset observed in the control group.	[86]

## 6. Packaging with Smart Indicator

Chameleons and cephalopods (e.g., octopuses) evolved color-changing abilities to adapt to their surroundings, while plants signal ripeness or stress through pigment shifts. Drawing inspiration from these natural mechanisms, scientists are designing smart food packaging that detects spoilage by changing color when exposed to biomarkers like ammonia, CO_2_, or pH shifts from decaying food. This bio-inspired technology offers a visual, real-time alert system to reduce waste and enhance food safety, transforming nature’s adaptive strategies into practical solutions for modern consumption [87,88,89]. Chemical indicators, such as phenol red, bromophenol red, bromophenol blue, bromocresol green, and methyl red, alongside natural pigment indicators like anthocyanins, carotenoids, chlorophylls, curcumin, and betaines, react with specific substances emitted during food spoilage to undergo color transformations, thereby visually representing the freshness of the food [90]. Such smart freshness indicators have demonstrated good application in the monitoring of a wide range of food products, such as shrimp [91], pork [92], mushrooms [93], and kiwifruit [94]. However, it is worth noting that nature’s chameleon continues to change color under complex conditions, and the color changes according to the contact object, which contrasts with the limitations of current food freshness indicators. The current intelligent indicator films for food often target only a single type of food, such as fruits or meats, and there is a lack of indicator films that are compatible with both. Additionally, there is insufficient attention and investigation into the durability of their indicating capabilities and their resistance to harsh environmental conditions.

**Figure 7:**
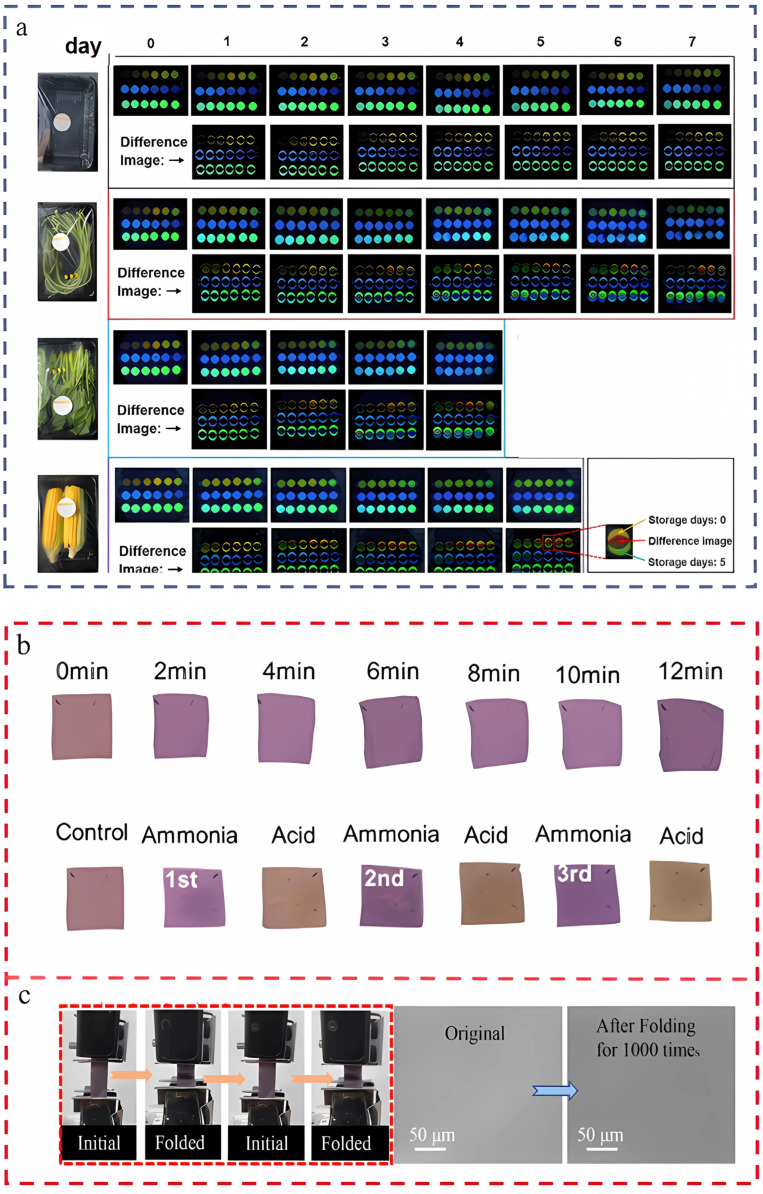
(**a**) Raw and difference images of fluorescence sensor arrays for blank control and three vegetables after different storage days [95]. (**b**) Images of films reacting with volatile ammonia (**top**) and reversibility of color change in films after repeated treatment with ammonia and acetic acid (**bottom**) [96]. (**c**) Fatigue testing of films and stabilization of microstructure after testing [97]. The above pictures have been reproduced with permission.

Fortunately, progress has been made by some researchers in overcoming these limitations. They created a vegetable freshness monitoring system by developing a pH-sensitive fluorescent sensor array that uses curcumin, puerarin, and fisetin. This sensor array was then combined with the ResNet50 deep convolutional neural network (DCNN) model, as shown in Figure 7a. The system was able to assess vegetable freshness non-destructively, in real-time, and intelligently with an accuracy of up to 98.58%, 97.15%, and 92.89% for yard beans, spinach, and sweet corn, respectively, which made an important contribution to the freshness indication of multiple types of vegetables [95].

In terms of indicator durability, Dong et al. used a layer-by-layer casting strategy to design a double-layer smart package, which not only has the function of color development, but also maintains sensitivity and stable visualization after at least three cycles (b), which is expected to be a model for durable indicators [96]. In addition, related researchers constructed a pH/moisture-sensitive chitosan/carboxymethyl cellulose adhesive layer containing anthocyanins on cellulose paper, which not only firmly immobilized the anthocyanins but also conferred superior structural stability and colorimetric functionality to the paper, with no significant change in the surface after at least 1000 folding cycles (c), and which indicated that the indication could be used to monitor the freshness of pork and blueberries [97]. Considering these research advancements, there are high expectations for the future development of food indicators. It is anticipated that products with enhanced durability and improved indication capabilities will emerge. Such food indicator labels should be capable of signaling the freshness of both meat and vegetables, and should also be able to undergo testing under harsher environmental conditions (such as −20 °C), thereby enabling a wider range of practical applications.

## 7. Self-Healing Packaging

The phenomenon of self-repair, as a manifestation of the excellent recovery ability of organisms to damage, widely exists in living organisms in the natural world. Research in regenerative biology has demonstrated that biological systems maintain inherent resilience through dynamic homeostatic mechanisms, as evidenced by the regenerative capacity of plant tissues following mechanical or environmental stress—a process optimized by evolutionary pressures to ensure species survival. Similarly, the regrowth of skin and muscle tissue after injury is a clear example of the role of self-repair mechanisms in living organisms [98,99]. This natural phenomenon is not only amazing, but also provides valuable inspiration for the development of human technology. In the field of science and technology, self-repairing hydrogels have been used in a wide range of applications, especially in human health monitoring [100], wound dressings [101], and disease management [102]. Similarly, this self-repair mechanism also brings new insights and perspectives to the field of food packaging design. In the application scenario of food packaging, the integrity of the package is crucial to maintain the freshness of the food. Once the package is damaged, its freshness will be significantly reduced, resulting in the food being more susceptible to the external environment and thus accelerating the spoilage process. In view of this, incorporating self-healing mechanisms into the design of food packaging is expected to slow down the deterioration rate and extend the shelf life of food products to a certain extent [103]. The ability of self-healing hydrogels to repair themselves is mainly attributed to their unique cross-linking mechanism. Covalently crosslinked hydrogels rely on dynamic covalent bonds in the network structure, such as disulfide bonds, imine bonds, reversible bonds in the products of Diels-Alder addition reactions, and bonds generated by reversible free radical reactions. These bonds can be broken and re-formed under specific conditions, thus restoring the integrity and function of the hydrogel. Non-covalently cross-linked hydrogels, on the other hand, form physical cross-links through non-covalent interactions such as electrostatic interactions, hydrophobic interactions, hydrogen bonding interactions, and host-guest interactions. These interactions are capable of spontaneous rearrangement and formation after damage, thus conferring self-healing properties to hydrogels [104,105].

Table 3 data analysis highlights generalized trends in the application of self-repair materials for food packaging. Systems relying exclusively on non-covalent interactions for damage recovery consistently show functional limitations, specifically manifested in three core areas. First, the self-healing process is typically slow, often requiring more than 15 min, as reported in the literature [106,107,108], whereas employing borate bonding as the main repair mechanism enables healing within a mere second [109]. Second, effective self-repair often necessitates additional conditions, such as heating or water supplementation, which increases operational complexity and cost. Third, even after healing, the mechanical properties of these hydrogels are often significantly compromised, limiting their applicability in scenarios requiring high strength or durability.

Notably, it is worth mentioning that many literature sources focus on the use of hydrogels for creating self-healing materials. However, applying self-healing hydrogels to food preservation presents even greater challenges and limitations. Many commonly used hydrogel components and self-healing agents, such as acrylamide, borax, and certain toxic photo initiators, are unsuitable for food-related applications due to safety concerns. This significantly narrows the selection of materials available for food-grade self-healing hydrogels, thereby constraining advancements in this field. Consequently, developing safe and effective self-healing hydrogel materials specifically designed for food preservation has become a critical issue in food science.

## 8. High-Strength Food Packaging

**Figure 8:**
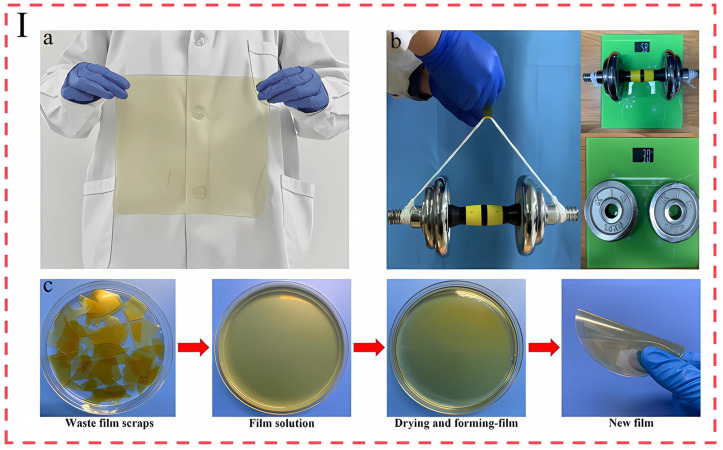

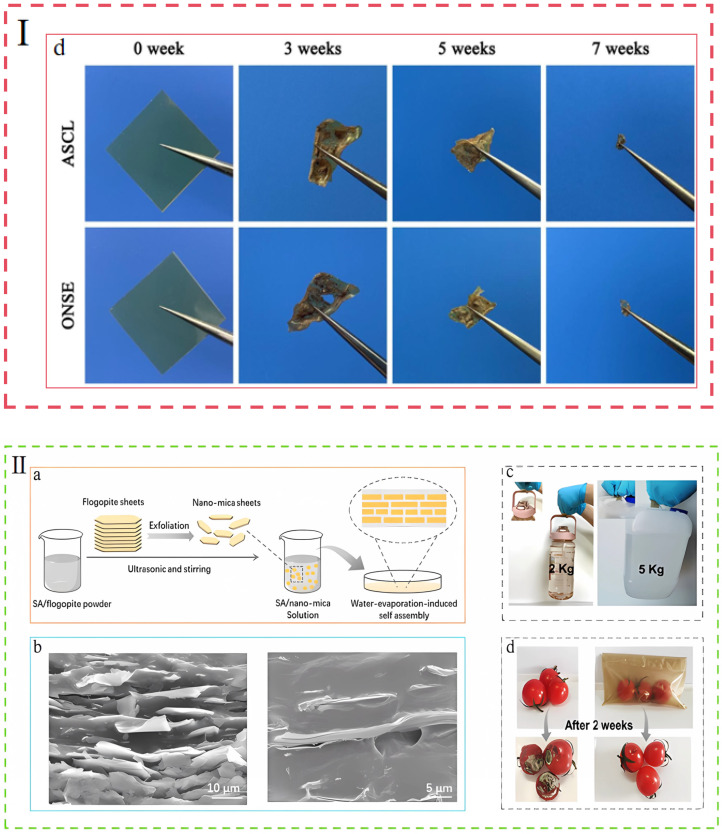
(**I**) (**a**) Appearance of the spider silk-inspired film. (**b**) Schematic of the film lifting a 5.8 kg weight. (**c**) Schematic of the re-formation of a new film after the shredded film is dissolved in hot water. (**d**) Degradability of the film [116]. (**II**) (**a**) Schematic of the pearl-layer-inspired film preparation process and the formation of bricks and mortar structure. (**b**) Scanning electron microscope image of the tensile fracture surface of the film (bricks and mortar structure formed). (**c**) Schematic of the bionic film lifting a 2 kg and 5 kg weight. (**d**) Photographs of the storage results of cherry tomatoes packaged in a composite film at different times (0 d and 14 d) and the control group [120]. The above pictures have been reproduced with permission.

The performance limitations of many food packaging machines significantly constrain their wide range of applications. However, the rich and diverse biological structures in nature provide a valuable source of inspiration for the optimization of material properties [114]. The high strength of spider silk originates from its dynamic energy dissipation structure of β-crystal-α-helix gradient co-structuring. In spider silk protein, alanine (which is arranged axially along the fibers to form a rigid skeleton, giving the material a high modulus) and the glycine motif induces the α-helix to form an amorphous zone with the flexible chain segments, which provides an elastic cushion through inter-chain slip and hydrogen bonding dissociation-rearrangement; when stretching, the preexisting hydrogen bonding promotes the rearranging of the amorphous zone into new β-crystals, so that the energy dissipation accumulates with the deformation gradient. During stretching, the preexisting hydrogen bonds induce the amorphous regions to reorganize into new β-crystals, so that the energy dissipation accumulates with the deformation gradient, and this dynamic synergistic mechanism of crystalline–amorphous realizes the balance between strength and ductility [115]. Inspired by this, Chang et al. constructed high-performance bio-based films based on dynamic nano-confinement effects using soy protein and tannic acid as well as hyperbranched polyester (Figure 8I(a)). Experimental characterization confirms that the film system exhibits an excellent tensile strength of 44.6 MPa and an ultra-high fracture energy of 44.7 MJ m^−3^, and its mechanical properties are further verified by static loading tests: a film specimen with a size of 12 mm (width) × 0.28 mm (thickness) can stably carry a weight of 5.8 kg with only elastic deformation (Figure 8I(b)). In the sustainability assessment, the waste film was completely depolymerized to a homogeneous solution after 12 h of hot water treatment at 90 °C. The recycled solution could be re-cast into a film that retained about 80% of its original properties (Figure 8I(c)), and the film was nearly completely biodegradable in 7 weeks (Figure 8I(d)) under natural environmental conditions [116].

Also of interest is the fact that the pearl layer of the pearl oyster shell exhibits excellent protective properties, with a “brick-mortar” hierarchical structure consisting of alternating layers of flat aragonite crystals (bricks) and polysaccharide–protein fibers (mortar), a biocomposite structure that is effective against predatory attacks and mechanical damage in complex environments [117,118,119]. Inspired by this, Wu et al. used sodium alginate as sand, natural mica nanosheets as bricks, and glycerol as plasticizer and cross-linker, and induced self-assembly by water evaporation, so that the mica nanosheets could be aligned isotropically and stacked in the in-plane direction instantaneously, and the films showed a brick and mortar-like structure similar to that of a natural pearl layer (II(a,b)). The resulting films were shown to have a tensile strength of 16 MPa and a thickness of 65 μm to withstand a weight of 2–5 kg (II(c)). After 2 weeks, the unprotected cherry tomatoes were soft and rotting, with molds appearing on the surface (II(d)). In contrast, the film-protected cherry tomatoes remained plump and fresh [120]. In order to show the application potential of biomimetic strategies in enhancing the mechanical properties of materials, Table 4 systematically combs through a variety of nature-inspired packaging material systems, and constructs a case library with reference value by integrating their biomimetic archetypes, material compositions, and mechanical property data.

For the enhancement of food packaging mechanical strength inspired by nature, it is crucial to consider both the absolute strength values and the improvements over the initial material properties. To provide a more intuitive understanding of the absolute values, here are some mechanical strength figures for commonly used food packaging materials. Low-density polyethylenhas has a tensile strength of 11.6 MPa and an elastic modulus of 171 MPa; high-density polyethylene demonstrates a tensile strength of 28.4 MPa and an elastic modulus of 625 MPa; polypropylene possesses a tensile strength of 20.23 MPa and an elastic modulus of 880 MPa; polyvinyl chloride shows a tensile strength of 24 MPa and an elastic modulus of 834 MPa; polystyrene has a tensile strength of 9.5 MPa and an elastic modulus of 760 MPa [101]. From the above data, it can be concluded that most of the food packaging developed by learning from nature exceeds the plastic-based packaging, while a small percentage of those that do not exceed it have significantly improved their mechanical properties from the original.

**Table 4:** Food packaging with enhanced mechanical properties.

Main Material	Bionic Object	Main Conclusion	References
Polylactic acid-coated mica nanosheets	Nacre	The film’s tensile strength saw a notable increase to 97 MPa, marking an 86.5% enhancement. The Young’s modulus also rose to 8 GPa, representing a 116% improvement. Additionally, its toughness climbed to 1.5 MJ/m^3^, a 25% boost, surpassing that of pure polylactic acid film by a considerable margin.	[118]
Oxidized nanocellulose, silver nanoparticles, MXene	Nacre	The film has a Young’s modulus of 4.4 GPa, which is 6.2 times higher than that of polyethylene, and a tensile strength that is 17.7 times higher, capable of supporting a weight of 1 kg, and is foldable and flexible.	[121]
Polypropylene carbonate, cellulose nanocrystals	Pearl layer	The maximum mechanical strength of the composite film was 12.6 MPa in the longitudinal direction, while the maximum tensile strength in the cross-section reached 9.1 MPa.	[122]
Catechol-functionalized chitosanPolyvinyl Alcohol	Mussel	The composite film exhibits a maximum tensile strength of 45.2 MPa and an elongation at break of 153%, representing an increase of 46.3% and 25.4% compared to pure polyethylene film, respectively.	[123]
Pullulan starch nanosheets	Vein network	The composite film demonstrates enhanced tensile strength, Young’s modulus, and toughness values of 51.05 MPa, 2.37 GPa, and 69.65 MJ/m^3^, respectively. These values represent significant improvements of 86.11%, 30.22%, and 223.36% compared to the pure Pullulan polysaccharide film. Moreover, these properties rival or surpass the strength of commercial plastic packaging films commonly utilized in the food industry.	[124]
Polylactic acid composite impregnated with hydroxyapatite nanocrystalline whisker	Pea pod	The yield strength of the composite material is 71.6 MPa, an increase of 54%. The elastic modulus of 2547 MPa was increased by nearly 72%, which was better than pure polylactic acid.	[125]
Collagen, nano zinc oxide, anthocyanidin	Pangolins	The film exhibits excellent tensile strength (78.64 MPa) and elongation at break approaching 50%.	[126]
Polyvinyl alcohol, cellulose micro-, nanofibers, calcium phosphate oligomers	Bone	The tensile strength of the bionic composite film was 145.57 MPa, and the toughness was 183.1 MJ/m^3^.	[127]

## 9. Future Outlook

### 9.1. Emergence of More Functional Bionic Packaging

In addition to the above applications, thermal management has a great deal to offer in the food sector. Inspired by polar bears, penguins, and other creatures, the future can be devoted to the development of new heat-insulating and cold-resistant materials to improve the insulation properties of packaging materials. In the field of anti-fog, natural structures such as the compound eyes of mosquitoes have become important reference objects for developing efficient anti-fog packaging. There is still great potential in mimicking natural water absorption mechanisms. For example, the water management strategies of plants such as cacti provide new perspectives for preservation technologies. Nature’s water-conducting mechanisms also aid in the development of water-absorbing pads. Furthermore, the highly adaptive sensory mechanisms observed in living organisms offer fresh insights for advancements in food packaging. Examples such as the trigger hairs of venus flytraps and the whisker systems of mammals highlight nature’s innovative sensory perception capabilities. Researchers are now mimicking these sensory functions to develop practical smart packaging solutions. Remarkably, the mechanical force sensing approach suggested by a group of researchers serves as a groundbreaking precedent for future scholars seeking to create sensory sensors that draw inspiration from natural mechanisms within the realm of food packaging [128]. In addition, based on the ubiquitous self-assembly and modularity principles observed in natural systems, the performance optimization and sustainable upgrading of food packaging materials can be achieved. By decoupling functional groups into independent modules (e.g., barrier units, antimicrobial modules, and reinforcement components), building standardized interfaces to achieve dynamic reorganization, and in situ repair of local damages through modular replacement, the service life of the material can be significantly extended, and the environmental load can be reduced. The self-assembly process utilizes intermolecular interactions to drive the precursors to spontaneously form an orderly structure, simplifying the manufacturing process while realizing the precise control of multi-scale structure, and providing an efficient path for large-scale production.

### 9.2. Multimodal Nature-Inspired Bionic Cling Film Design Prospects

Looking ahead, the design of biomimetic cling film is evolving steadily. It is no longer confined to mimicking a single biological feature; rather, it incorporates a range of excellent biological characteristics to make innovative technological advancements. To be specific, designers can enhance cling film’s durability by mimicking the pearl layer’s structure, improve its waterproofing by emulating the lotus leaf’s properties, and precisely regulate antimicrobial release by replicating plant stomata functions.

### 9.3. Combined with 3D Printing Technology

Compared with the traditional casting method, 3D printing technology in the field of film-making not only shows the ability of personalized design, but also predicts a huge potential in improving film-making efficiency and functional diversification. Based on the current research progress, 3D printing technology shows outstanding potential in the following aspects: first, the technology has the potential to realize direct and efficient reproduction of complex superhydrophobic textures on biological surfaces [129]. Natural textures such as lotus leaf papillae or shark skin shield scale morphology are expected to be accurately reproduced in a more concise process through 3D printing, which is expected to significantly improve efficiency and accuracy compared to traditional methods such as polydimethylsiloxane (PDMS) inversion molding and other multi-step transfer. Secondly, previous studies have demonstrated that 3D printing can precisely regulate the release of antimicrobial agents like cinnamon essential oil by adjusting the fill rate of printed structures [130]. In future research, 3D printing is expected to facilitate the fabrication of gas channels with tailored selectivity, thereby mimicking the ability of plant leaves to differentially regulate gas exchange. By integrating these channels into existing sustained-release packaging systems, researchers could augment their functionality with selective gas permeability. Specifically, the packaging could achieve sustained release of antimicrobial agents through adjustable fill rates, while simultaneously isolating oxygen and retaining carbon dioxide through membrane selectivity. This dual-action approach would emulate the dynamic aperture control and selective gas absorption and release of plant stomata, thereby mitigating oxidative degradation and extending food shelf life.

In summary, the inspiration of nature continues to drive the innovation of 3D printing technology in the field of food cling film production, while the development of 3D printing technology further broadens the boundaries of mimicking the natural structure, optimizing the material properties and realizing the advanced functionalized design, which are complementary to each other to promote the advancement of food bio-based packaging.

### 9.4. Overcoming Current Limitations

While nature-inspired innovations have driven significant advances in the field of food packaging, serious challenges remain: the use of toxic additives such as borax in self-healing designs and the use of certain metal–organic frameworks (MOFs) in controlled-release systems undermines food safety and restricts commercial viability, hence the need for non-toxic material alternatives; lab-centered research often ignores the dynamics of the food storage and transportation reality (e.g., temperature/humidity fluctuations). Finally, bionic packaging design faces industrialization challenges. Such packaging often requires elaborate designs, complex modifications, or is too costly to adapt to large-scale industrial production. Therefore, addressing these safety, real-world validation, and mass production deficiencies through material innovation, rigorous field testing, and streamlined production strategies is critical to moving food packaging to the forefront of safer, more efficient, and industrially viable packaging.

## 10. Conclusions

This paper provides a comprehensive analysis, summary, and comparison of nature-inspired bio-based materials for use as alternatives to traditional petroleum-based food packaging. Inspiration from nature has been harnessed to enhance the mechanical properties of bio-based materials, such as increasing their strength. These improvements tackle the critical limitations that have hitherto impeded their widespread application. In addition, bio-inspired properties, including self-healing capabilities, gas conditioning, and greater resistance to mechanical damage, as well as the development of superhydrophobic surfaces, have inspired the development of additional breakthrough properties of bio-based materials to replace plastic food packaging. For example, superhydrophobic surfaces play a critical role in extending the shelf life of food products by reducing moisture intrusion and helping to create environmentally friendly products such as paper straws, and superhydrophobicity is also helping to develop paper containers to replace plastic food containers and reduce food waste. Ultimately, these innovations are not only accelerating the transformation of food packaging but also providing a solid foundation for sustainable and practical solutions for food packaging. However, while affirming the positive impact of natural inspiration on food packaging innovation, we should also acknowledge its limitations. In the realm of self-repairing food packaging, for instance, while certain toxic reagents like borax can impart excellent self-repairing capabilities to packaging materials, their toxicity has restricted their practical application in food packaging. Moreover, challenges such as the lengthy duration of the self-healing process and the necessity of adding extra water have hindered the widespread adoption of this technology. Similarly, smart indicator packaging faces numerous hurdles in terms of durability, repeated color-change capability, and the integration of multiple indicator functions, necessitating further research and optimization. Finally, bionic packaging design faces industrialization challenges. Such packaging often requires elaborate designs, complex modifications, or is too costly to adapt to large-scale industrial production.

Looking forward, with ongoing advancements in technology and research, we remain hopeful that these obstacles will be gradually overcome. The future of food packaging holds the promise of being more intelligent, eco-friendly, and versatile. Nature, as a boundless source of inspiration, will continue to guide us towards more sustainable and efficient packaging solutions.

## Figures and Tables

**Table 3:** Self-healing food packaging.

Main Material	Self-Healing Mechanism	Repair Effect	References
Konjac glucomannan, Xanthan gum, gallic acid	Hydrogen bond	When water droplets contacted the scratches on the composite film, the scratches vanished entirely within 15 min, demonstrating the film’s remarkable self-healing capabilities. Additionally, this film extended the banana’s shelf life by over six days.	[106]
Sodium alginate, gluconolactone, whey isolate protein	Electrostatic force	Film scratches drip water after 20 min of self-healing, after repairing the mechanical properties of the composite film reaches more than 75% of the undamaged state, and can extend the banana freshness for 6 days.	[107]
Tamarind polysaccharide, polyvinyl alcohol	Hydrogen bond	The hydrogels were placed next to each other, and the edges blurred in 20 min and completely healed in 60 min. The hydrogel effectively retarded the rotting of red snapper filets at 4 °C and maintained the quality of the fillets.	[108]
Nanocellulose, polyvinyl alcohol, curcumin, borax	Borate and hydrogen bonding	The broken hydrogel film heals quickly within 1 s without external stimulation, and the film extends the shelf life of the fish to 9 days.	[109]
Sodium alginate, gluconolactone, wheat gluten	Electrostatic force	Scalpel scrapes the film and then drops of deionized water, the scratch heals itself in 60 s, demonstrating self-repairing properties while extending the freshness of the banana up to 7 days.	[110]
Hemicellulose, chitosan	Exogenous restoratives and ultraviolet light	When the film is scratched, the microcapsules break to release free radicals and polymerize and cross-link under UV light to achieve self-repair. This film keeps the quality of cashew nuts fresh for 60 days.	[111]
Sodium carboxymethyl cellulose, polyethyleneimine, polyvinyl alcohol	Possible dynamically reversible Physical bonds	The hydrogel film is self-repairing at room temperature, can support 500 g of weight after healing, and extends strawberry freshness for 7 days.	[112]
Oxidized alginate, carboxymethyl chitosan, anthocyanin	Schiff base linkages and hydrogen bonds	The severed hydrogel heals completely within one hour with a self-healing efficiency of approximately 93%, and Schiff base bonds have a significant impact on the self-repairing capability of the hydrogel.	[113]

## Data Availability

No new data were created or analyzed in this study. Data sharing is not applicable to this article.
